# Effects of Low Intensity Focused Ultrasound on Liposomes Containing Channel proteins

**DOI:** 10.1038/s41598-018-35486-1

**Published:** 2018-11-22

**Authors:** Meghedi Babakhanian, Limin Yang, Bryan Nowroozi, George Saddik, Lilian Boodaghians, Paul Blount, Warren Grundfest

**Affiliations:** 1 Department of Bioengineering, University of California, Los Angeles, CA 90095 USA; 2 Center for Advanced Surgical and Interventional Technology (CASIT), University of California, Los Angeles, CA 90095 USA; 3 Department of Electrical Engineering, University of California, Los Angeles, CA 90095 USA; 4 David Geffen School of Medicine, University of California, Los Angeles, USA; 5 Department of Physiology, UT Southwestern Medical Center, Dallas, TX 75390-9040 USA

## Abstract

The ability to reversibly and non-invasively modulate region-specific brain activity *in vivo* suggests Low Intensity Focused Ultrasound (LIFU) as potential therapeutics for neurological dysfunctions such as epilepsy and Parkinson’s disease. While *in vivo* studies provide evidence of the bioeffects of LIFU on neuronal activity, they merely hint at potential mechanisms but do not fully explain *how* this technology achieves these effects. One potential hypothesis is that LIFU produces local membrane depolarization by mechanically perturbing the neuronal cell membrane, or activating channels or other proteins embedded in the membrane. Proteins that sense mechanical perturbations of the membrane, such as those gated by membrane tension, are prime candidates for activating in response to LIFU and thus leading to the neurological responses that have been measured. Here we use the bacterial mechanosensitive channel MscL, which has been purified and reconstituted in liposomes, to determine how LIFU may affect the activation of this membrane-tension gated channel. Two bacterial voltage-gated channels, KvAP and NaK2K F92A channels were also studied. Surprisingly, the results suggest that ultrasound modulation and membrane perturbation does not induce channel gating, but rather induces pore formation at the membrane protein-lipid interface. However, in vesicles with high MscL mechanosensitive channel concentrations, apparent decreases in pore formation are observed, suggesting that this membrane-tension-sensitive protein may serve to increase the elasticity of the membrane, presumably because of expansion of the channel in the plane of the membrane independent of channel gating.

## Introduction

The treatments of neurological and psychiatric diseases, such as Alzheimer’s, Parkinson’s and epilepsy, as well as neuropathic pain management are currently limited to pharmacologic or invasive surgical strategies. While pharmacologic treatments can be designed to target specific neurotransmitters, they lack the regional selectivity that some device interventions can give. In addition, adverse effects are often associated with pharmacotherapy as well as concerns about drug metabolism and clearance in individuals with compromised hepatic and renal function^1^. On the other hand, neurosurgical interventions such as resections and Deep Brain Stimulation (DBS), now established clinical procedures and used in clinical studies for a myriad of neurological applications, can target specific regions of the brain but are invasive and have associated morbidity risks^2–4^. Novel transcranial magnetic stimulation has the advantage of being completely noninvasive and the ability to focus on deep brain structures, but it affords low spatial resolution^5^. Thus, there exists an acute need for an affordable, non-invasive neuromodulation intervention that can precisely target deep brain structures *in vivo*. To address these limitations, there has been increased interest in using focused ultrasound (FUS) as a strategy for a noninvasive neuromodulation that can be targeted to specific brain regions.

Ultrasound application as both a reversible neural suppressor and activator was not explored until the 1950s^6,7^*. In vitro* studies revealed that FUS can effectively stimulate both neurons in culture as well as induce a short-latency excitatory response in a rodent brain-slice assay^8,9^. These findings were further investigated in many small and large *ex vivo* animal models including rodents^8–13^, nonhuman primates^14,15^, and humans^16^. Though characterized by a somewhat variable success rate, these studies appear to show compelling *in vivo* evidence of the neuromodulatory capabilities of FUS, leading investigators to believe that FUS may be a candidate for transcranial neuromodulation for conditions like Parkinson’s disease and Epilepsy. The variability observed between studies and the limited success may be a result of the lack of knowledge regarding (1) the mechanism underlying neuromodulation and (2) effective system parameters that successfully stimulate, or suppress, nervous activity.

Currently, the prevailing hypothesis explaining the neuromodulatory capacity of FUS suggests that pressure transmitted to the tissue creates conformational changes in the lipid membrane due to its elastic characteristics, causing modulation of protein channels and mechanoreceptors embedded within the membrane. This modulation of protein channels is then thought to have effects on cellular excitability, action potential variation, and neurotransmitter release or uptake^9,17^. As a result, several studies investigated the effect of ultrasound on ionic flux using ion-specific dyes^9,18^. Because of the ion permeation observed, channels have become candidates for the conduits for the ion fluxes observed. The authors of one of these studies have speculated that the US-induced mechanical forces in the membrane modulates channel activity^10^. Indeed, several mechanosensitive (MS) channels have electrophysiologically been shown to be directly gated by membrane tension^19–23^, and even several channels normally gated by voltage or ligands have been shown to be modulated by forces in the membrane^24–27^. If this notion is true, one would anticipate that MS channels that are gated directly by membrane tension would be extremely sensitive to FUS.

*E. coli* mechanosensitive channels have been used in multiple studies to investigate the protein-lipid interaction and how tension in lipid bilayers can induce changes in protein conformation. One of these proteins is the Mechanosensitive Channel of Large conductance, MscL. This channel has been shown to directly sense membrane tension rather than membrane curvature or the pressure across the membrane^28,29^. In addition, MscL has the largest known gated pore, estimated to be greater than 30 Å in diameter^30^, thus allowing relatively large molecules to flux through its pore^31,32^. Therefore, the effects of membrane tension on protein conformation have been more easily studied by physiological and biophysical approaches. These properties make MscL a great paradigm for investigating the mechanical influence of low intensity and low frequency ultrasound on cells and its structural changes at the molecular level.

Here we have performed studies to investigate the effects of low intensity focused ultrasound (LIFU) stimulation and varying parameters on MscL using a simplified *in vitro* proteoliposome model. Our set up enables us to study the effects on a pure system containing only the protein and lipid, without the need to take the cellular cytoskeleton and other structures into consideration. Our liposomes include either the mechanosensor MscL, or one of two other channels that are not thought to be mechanosensitive, NaK2K F92A and KvAP. We evaluated different acoustic intensities that are potentially the most clinically relevant for *in vivo* animal and human applications, i.e. those shown successful in previous *in vivo* ultrasound modulation experiments and aligned with current FDA regulatory limits on clinical ultrasound imagers^33^. Our results suggest that LIFU can modulate cell membranes and allow efflux through pores made at the protein-membrane interface rather than gating the channels themselves. In addition, the MscL mechanosensitive channel, when reconstituted at high concentrations, actually inhibits this pore formation, presumably by acting as an elastic, absorbing the forces in the membrane without actually undergoing full channel gating.

## Results

### Acoustic Beam Properties

As a precursor to liposomal experiments, the acoustic field was characterized. This was performed for a single-element focused transducer, operating at 0.5-MHz frequency, using a calibrated hydrophone in two different settings and varying parameters. Initially, three-dimensional shape of the beam was characterized inside an acoustic tank. The beam focus was measured to be at 29 mm from the transducer with a full width at half maximum (FWHM) diameter of 3 mm. Based on the measured free space beam shape and FWHM cross-sectional dimensions, the beam measurements were translated into a 96 well plate.

The acoustic pressure measured inside a 96 well plate was around 140 kPa that corresponds to 83 kPa inside the tank. Conversely, the intensity was varied by altering the duty cycle (DC). As we raised the DC from 60% to continuous wave (CW), the I_spta_ value increased from 122 mW/cm^2^ to 194 mW/cm^2^ inside the tank and 240 mW/cm^2^ to 394 mW/cm^2^ inside the well. As expected, we noticed a higher I_spta_ and pressure value when the measurements were performed inside the well plate compared to the tank.

### LIFU Parameter Optimization

To detect permeation from liposomes, we utilized a simple system previously used by us and others^34–36^. Briefly, we loaded liposomes with the dye calcein. One of the properties of this dye is that at high concentrations it is self-quenching. Hence, when encapsulated within the liposome, the dye shows little fluorescence. However, when fluxed from the liposome, the concentration of the dye goes below the concentration needed for self-quenching, and the fluorescence can easily be measured. The fluorescence increase upon LIFU treatment was normalized by that after Triton X-100 treatment, which completely lyses liposomes, to yield percentage calcein efflux for comparison between different experimental groups.

Our first objective was to determine the effective ultrasound parameter set that is necessary to make the assay the most sensitive. To perform this, we initially investigated the LIFU stimulation duration effect (0, 5, 10 and 20 min) on membrane modulation and permeability, independent of channel reconstitution. We observed that a 5 min LIFU duration yielded very small or no efflux (~3%). However, our results from 10 and 20 min duration of LIFU stimulation in liposomes showed about 5% and 17% efflux respectively. In sum, the 10 min duration of LIFU stimulation was the shortest time period that gave consistent and statistically significant differences and was therefore used in all subsequent experiments.

**Figure 1:**
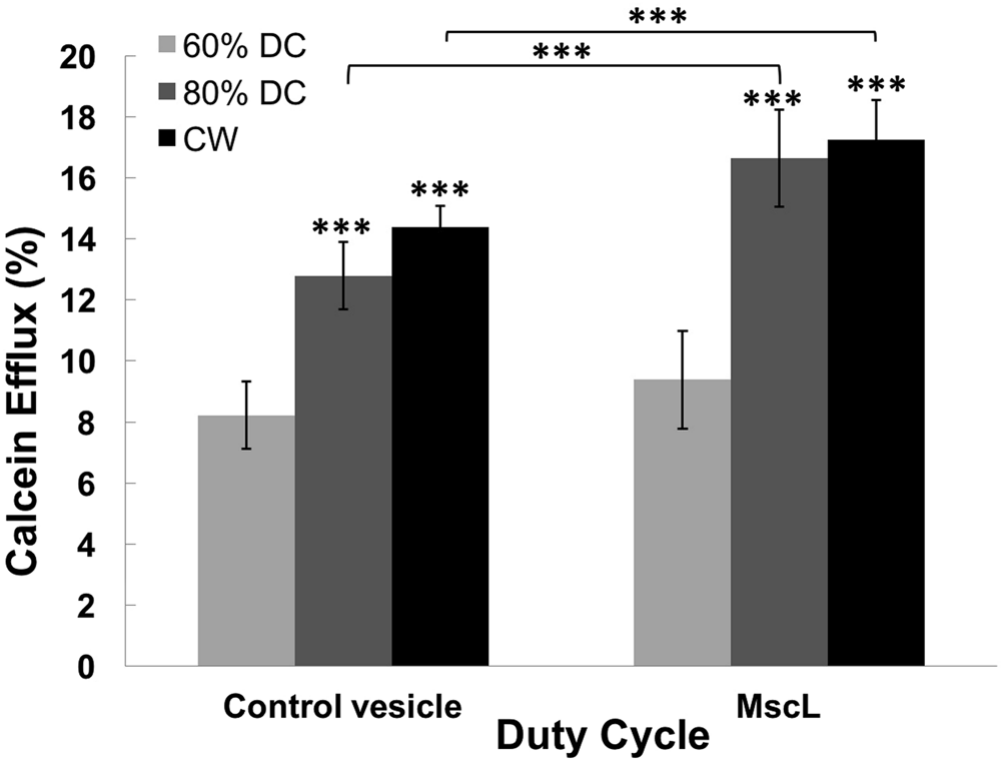
LIFU stimulation duty cycle effect studies involved varying duty cycle (60%- light grey, 80%- Dark grey and CW-Black) within three groups (Control vesicle or MscL); analysis using mixed effects linear regression **p-value < 0.001. (Control vesicle group: 60% DC 8.6 ± 1.1 (n = 30), 80% DC 13.1 ± 1.1 (n = 30), CW DC 14.9 ± 0.7 (n = 85); MscL group: 60% DC 8.4 ± 1.6 (n = 33), 80% DC 15.3 ± 1.6 (n = 28), CW DC 17.6 ± 1.3 (n = 90)).

To further define the effective parameter set, we studied DC as our second parameter, which reflects variation in intensity (I_spta_). We varied the pulse DC from 60% to a CW but kept frequency (0.5 MHz), input voltage (28 dBm) and stimulation duration (10 min) constant. This set of experiments was conducted using vesicles with and without MscL channels. For the vesicle with MscL, the protein concentration was kept constant at the highest concentration during the DC studies for simplicity (380 pmol of MscL/mg of lipids). A mixed effects regression statistical model was used to compare the higher DC values to the 60% DC per each experimental design. We found that 60% DC was sufficient to induce the efflux of calcein from the vesicles, and that slightly larger fluxes were observed as the stimulation acoustic intensity was increased from pulsed wave to continuous wave (Fig. 1).

We next compared calcein efflux between vesicles with and without MscL for each duty cycle. We consistently observed a significant increase between vesicles with and without MscL channels (Mixed effect linear regression p-value = 0.001), except at the 60% DC. The efflux gap between the vesicles containing MscL versus those that do not is the greatest at the higher DCs. Thus, it appears that 10 min LIFU duration and CW conditions yielded the best results, and were therefore used in all subsequent experiments below.

### Effect of Channel Protein and their Concentration on LIFU-induced Proteoliposome Efflux

To understand the effect of LIFU on protein-membrane modulation, we reconstituted liposomes with different protein channels at different protein-lipid ratios. Since ultrasound treatment may generate mechanical stimuli, we chose bacterial mechanosensitive channel of large conductance, MscL, because this channel is well known to be gated by membrane tension^28^. As controls, we also included channels not thought to be gated by mechanical force, show preferences for potassium permeation, and should not allow flux of the calcein through their pore. These were the *Bacillus cereus* voltage gated NaK2K F92A channel mutated at residue 66 from aspartate to proline, 68 from asparagine to glutamate and 92 from phenylalanine to alanine, which is reported to be selectively permeable to potassium ions^37,38^, and the *Aeropyrum pernix* voltage-gated potassium channel^39^, KvAP.

**Figure 2:**
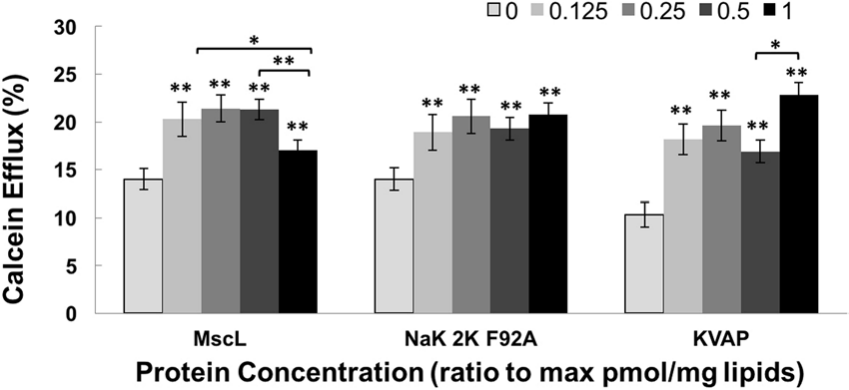
Calcein efflux from proteoliposomes reconstituted with different concentrations of MscL, NaK2K F92A and KvAP treated with and without ultrasound. Lightest grey bar line per group (MscL, Nak2K F92A, KvAP) shows control vesicle (no-protein) % efflux in respond to LIFU stimulation. Black bar line per group presents % efflux for the highest protein concentration level possible per group (MscL 380 pmol/mg lipids, NaK2K F92A 467 pmol/mg lipids, KvAP 77 pmol/mg lipids). The three grey bar lines between the lightest grey and the black bar represent liposomes reconstituted with 0.125, 0.25 and 0.5 of the highest concentration of the corresponding protein. Each well was modulated once at a time, for 10 minutes. As protein concentration increased, significant increase in efflux was noticed among all three groups except the highest MscL concentration (n values from left to right equals, MscL group: 85, 90, 49, 36, 17; NaK group: 56, 55, 22, 15, 13; KvAP group: 26, 30, 18, 18, 18) (Mixed effect linear regression, *p < 0.05, **p < 0.01).

Different protein channel concentrations were reconstituted into liposomes to study concentration effect on efflux. The maximum feasible concentration possible per channel type was defined empirically (MscL 380 pmol/mg lipids, NaK2K F92A 467 pmol/mg lipids and KvAP 77 pmol/mg lipids); at higher concentrations, the yield of proteoliposomes drastically diminished, as indicated by smaller liposomal pellets as well as decreased total load of calcein assayed after Triton-X100 treatment. These maximum concentrations of protein, as well as diluted concentrations (0.5, 0.25 and 0.125 of the maximum) were reconstituted in liposomes and LIFU-induced calcein effluxes from liposomes containing these different MscL concentrations were statistically compared to the calcein efflux of the no-protein control liposome group (Fig. 2). The no-protein liposome group had the lowest efflux with the efflux percentage rising as the MscL protein concentration increased; the one exception was the liposomes with the highest MscL concentration, which showed a decrease at the highest concentration (21.3 ± 1.3% (n = 49) at the 0.5 protein concentration, but only 17.0 ± 1.1% (n = 90) at the maximal concentration). As a control for protein absorption of ultrasound energy, we also tested calcein efflux from no-protein liposomes with bovine serum albumin added to the solution. No significant difference was observed with or without this control protein (23.11 ± 3.17 and 22.92 ± 1.71 for LIFU-treated no-protein liposomes with and without the presence of bovine serum albumin in the solution, respectively (n = 3)).

We performed the identical experiment on liposomes containing the voltage-gated bacterial channels NaK2K F92A and KvAP. Surprisingly, liposomes harboring these channels showed a similar trend of efflux increase as that seen for the MscL channel containing liposomes, with the exception that the liposomes containing the highest channel concentrations did not show decreased efflux (Fig. 2).

Hence, calcein efflux increases as a function of higher acoustic intensity, but the efflux isn’t selective - all three membrane protein channels lead to an increase in calcein efflux. Neither of the voltage-gated channels are thought to form a pore large enough to pass the relatively large calcein molecule (about 10 Å). In addition, both channels show cationic selectivity, while calcein is an anion with two negative charges. Therefore, it seems unlikely that calcein is passing through the KvAP or NaK2K F92A channel pores. Given that the calcein flux is channel independent, a more likely explanation is that the perturbations in the membrane lead to large pores that form at the protein-lipid interface.

### The Effects of Lipid Tension Induced by Lysophosphatidylcholine (LPC) on Calcein Efflux

We reasoned that if LIFU forms large pores at the protein-lipid interfaces simply by adding tension to the membrane, and ions, including the dye calcein, can cross the membrane through these pores, then adding tension to the membrane by other means may yield similar results.

**Figure 3:**
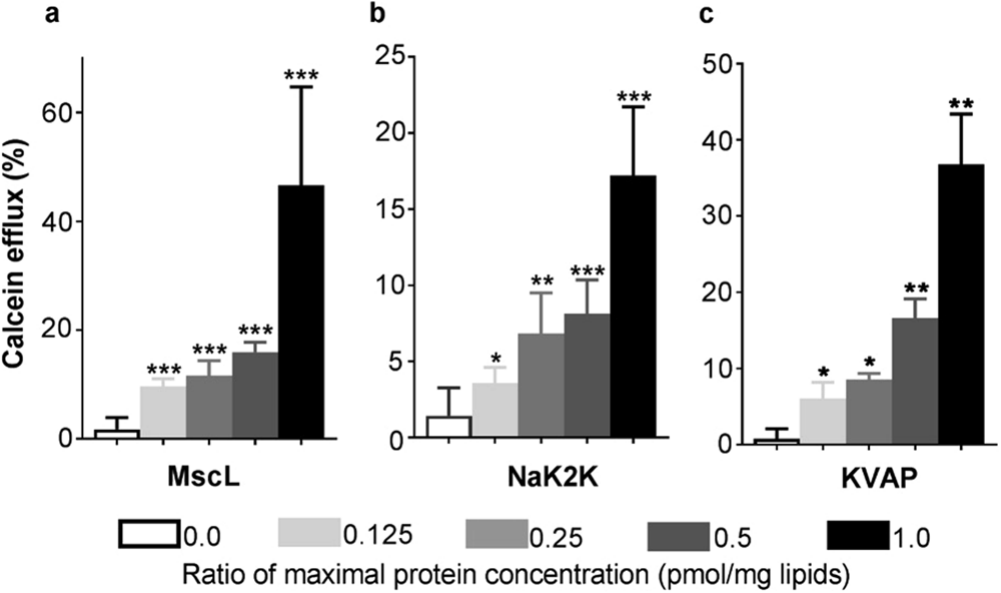
LPC induced calcein dye efflux from liposomes reconstituted with different protein channels: MscL (**a**), NaK2K F92A (**b**), KvAP (**c**) with varying protein concentrations per protein channel group. Calcein efflux increases for all three channels as the protein concentration increases. There is a significant difference (*P < 0.05, **P < 0.01, ***P < 0.001) between sham (no-protein liposomes) and proteoliposomes per protein channel group based on Mann-Whitney test. For no-protein and each protein concentration n equals 16, 5, 8, 12, 16 (**a**, MscL) 9, 5, 5, 7, 9 (**b**, NaK2K F92A) and 5, 4, 4, 5, 5 (**c**, KvAP).

LPC is an amphipath that contains only a single fatty acid chain, but a normal sized headgroup; thus, addition of LPC to the membrane changes the membrane lateral pressure profile and adds stress within the membrane. Lysolipids such as LPC, as well as other amphipaths that intercalate into the membrane asymmetrically, have been shown to activate mechanoproteins, including MscL^40^. We therefore determined the effects LPC in our calcein/liposomal system at varying concentrations of purified MscL to determine its effects on calcein flux. As expected, we found a LPC induced calcein flux that was MscL-dependent (Fig. 3a).

We then performed the same experiment using the two bacterial voltage-gated channels, KvAP or NaK2K F92A, which have not been shown to be mechanically sensitive. Liposomes containing either of these voltage-gated channels also effluxed calcein in a channel-concentration dependent manner (Fig. 3b,c).

Collectively, these data show that calcein fluxes out of liposomes in a channel independent manner with either LIFU or LPC stimulation. It does not appear to matter which channel is expressed. Both stimuli are thought to add tension in the membrane; membrane proteins, which are more rigid and less dynamic than membranes may resist these tensions. Because of the size of calcein and the properties of the channel pores, perhaps the simplest explanation is that for both, LIFU and LPC, calcein fluxes from non-specific pores formed at the protein-lipid interface rather than by gating of the channels.

## Discussion

Several *in vivo* studies have revealed effective ultrasound stimulation of nervous tissue resulting in measurable ion fluxes and action potentials^9,11,14,16,41^. Indeed, a recent study even shows MscL overexpressed in neuronal cells has been reported to be activated by acoustic force^42^, strongly suggesting that when cytoskeleton and extracellular matrix are present, ultrasound can indeed induce membrane tension. Despite these recent successes, there remains a gap in our knowledge of the mechanisms underlying *in vivo* stimulation and the best ultrasound spatial temporal parameter to induce such stimulation. Different acoustic parameter sets can induce a variety of biological effects depending on cell type and tissue structure^9,11,43^. As an initial attempt to address these gaps in our knowledge, we explored the mechanism of LIFU sonication by narrowing down our studies to a specific LIFU parameter set and a simplified *in vitro* proteoliposome system. Our studies use only defined lipids and the MscL channel, for which protein-lipid interactions have been well-characterized; thus, we avoided the effect of other living-cell components such as the cytoskeleton. Additionally, we studied non-MS channels, initially thought of as controls, and compared these data to those obtained for the MscL channels to confirm whether efflux is mediated through the MscL channel pore in this simplified system.

The LIFU parameters chosen for these studies were based on previous successful *in vivo* ultrasound modulation studies and FDA safety regulations for diagnostic imaging (under FDA limit of I_spta_ = 720 mW/cm^2^) (AIUM Clinical Standards Committee 2004). Low ultrasound frequency was found to be more favorable in transcranial ultrasound stimulation due to lower acoustic beam attenuation and aberration through skull bone^44^. According to these results and the successful transcranial neuromodulation studies conducted using 0.5 MHz in small animal and human models, we chose 0.5 MHz frequency for our studies^8,11^. The intensities applied in our studies are considered to be at the lower range of intensities found effective in previous *in vivo* transcranial ultrasound neuromodulation studies^11,13^. We understand that the use of continuous wave and submersion of the hydrophone in this study may have caused reverberation within the small well volume. However, the intensities reported at the focal point inside the well plate are still considered safe and fall in the low intensity range for acoustic neuromodulation.

Earlier *in vivo* LIFU modulation studies were conducted using pulsed stimulations, emphasizing that pulsed modulation is more effective than CW stimulation^10,13^. However, *in vivo* studies showed that LIFU stimulation was found more effective as a function of both acoustic intensity and duration^11^. Our findings that longer durations of stimulation, and CW over pulsation, has stronger effects on the protein membrane perturbation agrees with previous results^11^.

Based on the proteoliposome modulation results using our LIFU system, we observed an increase in calcein efflux from liposomes reconstituted with MscL. We initially thought the increased efflux was due to MS channel activation induced by LIFU, but similar efflux values were measured in non-mechanically stimulated channels exposed to ultrasound. Knowing that the calcein dye cannot traverse NaK2K F92A and KvAP channel pores due to their size restrictions and ion selectivity, channel gating was ruled out. Pore formation through the membrane, modeled by Krasovitski^17^, or protein-lipid interface interruption mechanisms, may explain the increase in efflux behavior when any of the three channels were reconstituted into our *in vitro* system. Krasovitski’s proteoliposome simulation model called “bilayer sonophore”, or BLS, explains the cellular membrane’s ability to absorb LIFU mechanical energy and transforms it into expansions and contraction of the intramembrane space. The BLS simulation model showed that the extremity of bio-effects on the cell membrane depends on different ultrasound parameter exposure as well as the maximum area strain-absorbing ability of the leaflets. According to this theory, the bio-effects may vary from delicate and reversible excitation of the cell membrane to pore formation and even damage to membrane proteins and/or cytoskeletal fibers^17^. As the bilayer leaflets separate, the proteins inserted within the membrane will resist this expansion – α-helixes cannot easily expand in this manner. Thus, in theory, insertion of more rigid membrane proteins, such as the channels we tested, may lead to additional strains in the leaflets, making lipid pore formation at or near the protein-lipid interface more probable; it is as yet unclear if this also plays a role in some *in vivo* systems.

It is possible that this phenomenon in proteoliposomes could be exploited: transmembrane peptides could be designed to accentuate this pore formation for the purpose of targeted drug delivery. Liposomes, such as the ones we designed, tend to lodge in cancerous and inflamed tissues, but do not easily release their contents^45–47^. To enhance release of drugs loaded within liposomes containing the transmembrane peptide, LIFU may also be targeted to the problematic or diseased area. In this way, one could conceivably design a targeted and triggered drug release device.

Despite the observation that all three channels increased the efflux from vesicles, MscL channels appeared to react differently to the LIFU stimulation when compared to the non-mechanically stimulated protein channels. Specifically, vesicles containing the highest concentration of MscL showed a reduction, rather than increase, in calcein efflux when compared to liposomes with lower protein concentrations. Previous studies have suggested that the MscL channel may expand in the plane of the membrane prior to pore opening, forming what has been coined as a closed-expanded state of the molecule^48,49^. This expansion of the protein may be more exaggerated when compared to other channels primarily because of the extremely large pore that opens (about 30 Å in diameter). One interpretation of our data is that the MscL protein, by achieving the closed-expanded state, acts as a tension-spring, relieving some strains within the membrane. Indeed, there is precedence for such an idea. Boucher and colleagues previously explored how MS protein channels with stretching abilities, also referred to as membrane “spandex”, can maintain bilayer tension within a particular range by what was called closed-closed expansion^50^. This two-state (expanded/contracted) simulated model was inspired and designed based on characteristics and expression levels of bacterial MscL channels. The model tested the closed-closed expansion en route to opening, or preopen expansion states in the activation path, and overexpression in the bacteria, which act as stretch-tension buffers to avoid unnecessary opening of MscL channels^50^. MscL preopening expansion transition has been predicted to expand as far as 80% its open area prior to gating and thus behave as tension-damping spandex components^51^. Theoretically, the mechanosensitive channels, under appropriate conditions, could dampen the acoustic energy transformation onto the intramembrane spacing by conformational expansion leading to slow membrane tension relief. Therefore, assuming that LIFU induces tensions within the membrane as indicated by a recent study with MscL^42^, our findings appear to provide the first experimental evidence for Boucher’s “spandex” model.

## Conclusion

Our results suggest a new paradigm for the mechanisms underlying ultrasound stimulation. We find an increase in efflux of calcein through proteoliposomes when any of three membrane protein channels are reconstituted into them. Our data are not consistent with an increased efflux due to channel opening. Instead they suggest a mechanism in which “stiff” membrane proteins thwart the dynamics of LIFU-stimulated membranes, adding additional stresses, and ultimately pores are more likely to be formed at or near the protein-lipid interface. MscL, at its highest concentration, partially inhibits additional calcein release; this is presumably because of the ability of this channel to expand within plane of the membrane, and thus relieve some of the tension. These unexpected results add a new dimension to the mechanisms underlying LIFU stimulation of biological neuronal tissue.

## Online Methods

### Experimental Design

Four sets of liposomes were used to study the effect of FUS on ion channel gating and membrane tension. Unlike in living cells, the confounding effects of cell components such as cytoskeleton are not an issue in these simplified *in vitro* experiments. First, we generated liposomes with no protein channels. The next two liposome sets contained reconstituted NaK2K F92A or KvAP potassium channels, which are not reported to be gated by mechanical stimuli. NaK2K F92A is a mutant of the NaK channel that is K^+^ selective that has been well characterized^37,38^. Finally, we used MscL to compare LIFU effects on mechanical sensitive channels to non-mechanical sensitive channels, no channels, or bovine serum albumin.

### Liposome Formation, Protein Channel Reconstitution and Calcein Dye Loading

The protocol for MscL^28^, NaK 2K F92A^38,52^ and KvAP^53,54^ purification and reconstitution has previously been established^34–36,55^. The lipid used was composed of 1,2-Dioleoyl-sn-glycero-3-Phosphatidylcholine (DOPC), cholesterol, and 1,2-distearoyl-sn-glycero-3-phosphoethanolamine-N-[amino (polyethylene glycol)-2000] (DSPE-PEG 2000) in a molar ratio of 70/20/10. Lipids were dried and rehydrated with 50 mM calcein to form liposomes, which were then saturated with Triton-X100 and mixed with purified protein channels. The reconstitution of protein channels was completed upon removal of the detergent by Bio-Beads^TM^ SM-2 adsorbent (Bio-RAD) and separation of proteoliposome from the free calcein was performed by running the sample through a size exclusion column. The resulting individual liposomes have an average diameter of about 100 nm, as described^35^.

Calcein self-quenches at high concentrations but fluoresces at lower concentrations, i.e. if it is fluxed out of the loaded liposomes. This assay is used here since it allows on-site detection and it quantifies changes in liposome membrane permeability upon exposure to ultrasound and other stimuli.

### Ultrasound System and Set Up

To investigate the mechanism underlying ultrasound stimulation we used a single element commercially available focused transducer (V301-SU, Olympus NDT, Waltham, MA). This system was designed to accommodate culturing plates and well plates of different sizes. The bottom of the plate was exposed to the transducer through a coupler. Water is used in our system to couple the acoustic waves from the transducer to the bottom of the well plate. The acrylic cylindrical tube called the coupler was fitted on the transducer which created a sealed water filled acoustic chamber for the acoustic wave irradiation. Therefore, the coupler created a sealed water filled sonication chamber for the ultrasound irradiation. The coupler’s height was chosen such that the suspended cell volume inside the well was positioned at the focus of the transducer.

Sinusoidal waveforms were generated using a 20-MHz function generator (33220A, Agilent, Santa Clara, CA). The signal was amplified using a Minicircuit’s RF amplifier (ZHL-32A, Minicircuit, Brookly, NY) with a gain of 25 dB, powered by an Instek power supply (PST-3201, GW Instek, New Taipei City 236, Taiwan). The output waveform was electronically coupled using a custom made matching network before being sent to the focused ultrasound transducer with a center frequency of 0.5 MHz, a diameter of 25.4 mm and focal length of 30 mm. The waveform used in our experiment consisted of the following parameters: the frequency of the transducer was kept constant at 0.5 MHz, Pulse Repetition Frequency (PRF) was kept at 1 KHz, Number of Cycles (NOC) and voltage input were both varied. However, since only the highest voltages resulted in sufficient results, they are the only values reported.

### Acoustic Field Mapping Inside Tank and Well Plate

The ultrasound beam characterization was conducted using a needle hydrophone (HNR-1000, Onda Corporation, Sunnyvale, CA) submerged both in a standard acoustic field mapping system filled with deionized water and a culturing well plate solution. Originally, the acoustic performance of the transducer was measured in a precision tank called Acoustic Intensity Measurement System (AIMS, Sonora/Unisyn, Longmont, CO) using the needle hydrophone. AIMS is a precision scanning system mounted on a water tank that allows measuring and mapping of acoustic fields in liquids. The water tank is a common way of performing acoustic characterization of a system to mimics tissue properties. The hydrophone was mounted on a three-axis precision stage and the transducer was placed on a stationary stage with angular positioners for alignment purposes. Acoustic field scans in three-dimensions were performed to find the focal point. After the hydrophone and transducer were aligned, a Z-axis scan was performed in increments of 0.02 cm to find the focal point. Next, raster scans were conducted at the focal point with a Field of View (FOV) of 3 × 3 cm to measure the x-y beam map at the focal point. At the focal point, more measurements were acquired by varying the duty cycle (DC) and keeping the remaining parameters constant.

Subsequently, we conducted acoustic field measurements inside the 96 well plate by submerging the Onda hydrophone inside the small well and measured the intensity at its focal point. The arrangement of the well plate with respect to the transducer mimicked the experimental set up explained in the next section.

Also, the set up used in this 96 well plate beam characterization was repeated during the subsequent experiments, i.e. the hydrophone was submerged inside the well during *in vitro* stimulation testing to replicate a similar acoustic environment to the settings used during characterization (including potential reflection due to the hydrophone).

### Experimental Protocol for Ultrasound Treatment of Liposomes

As mentioned above, the experimental set up included a transducer coupled to the bottom of a 96 well plate via DI water. An appropriate volume of vesicles or proteoliposomes was added to the vesicle buffer in 2 wells of a clear bottom 96-well plate to achieve a final volume of 200 µL per well. Number of vesicles were kept similar in both wells which results in a similar fluorescent signal value after Triton X-100 treatment. One well was kept as a control and the other was applied with LIFU from the bottom of the plate well. The wells were chosen to be at least 5–6 well distances from each other to avoid potential cross talk. Prior to LIFU treatment, baseline fluorescence was recorded at 538 nm with an excitation at 485 nm using a Fluoroskan Ascent plate reader (Thermo Scientific Inc., Waltham, MA, USA).

Next, the sample well was treated with ultrasound using different parameter sets. First, control vesicles were treated with CW ultrasound at room temperature for 0, 5, 10, and 20 min durations. We obtained preliminary data for this sample set since only a few data points were collected for the 20 min duration. However, the results reflected a clear trend in duration effect on liposome modulation. Using the shortest and consistently effective duration (10 min), duty cycle (DC) effect was studied using control-vesicles, and MscL proteoliposomes by varying DC between 60%, 80% and CW. The minimum and maximum average calcein efflux range was defined based on these parameters and the parameters that resulted in the maximum efflux were used for the rest of the experiments.

Fluorescence was measured after ultrasound modulation using the same plate reader. Finally vesicles were lysed at the end by adding 10 µl of 10% Triton X-100 to determine the total calcein fluorescence levels in liposomes. All signals were normalized to the total florescence values after Triton treatment.

In addition, temperature changes were monitored before and after each LIFU treatment inside each well – no change in temperature was observed. Also, the absence of air bubbles was confirmed inside the coupler and the wells.

### Characterizing the Effect of Different Protein Concentrations on Ultrasound-Induced Membrane Permeability

We also evaluated the effect of different protein concentrations on ultrasound-induced changes in membrane permeability. The liposomes had different tolerances for the three channels tested, beyond which the liposome yield was drastically reduced. Channels were reconstituted into liposomes to the maximum feasible concentration possible per channel type (MscL 380 pmol/mg lipids, NaK2K F92A 467 pmol/mg lipids and KvAP 77 pmol/mg lipids). We then diluted the protein amounts to 0.5, 0.25 and 0.125 of the highest concentration per protein channel and reconstituted them into liposomes. Each well was modulated one at a time and in the following order: no protein vesicle, MscL, NaK2K F92A, KvAP, 0.5MscL, 0.5NaK2K F92A, 0.5KvAP, 0.25MscL, 0.25NaK2K F92A, 0.25KvAP, 0.125MscL, 0.125NaK2K F92A and 0.125KvAP. This order was repeated many times per experiment/day. These protein concentration range studies allowed us to observe the effect of channel concentrations on membrane permeability induced by ultrasound.

### Verification of the Presence of the Protein Channel and their Behavior

To verify protein channel presence and their gating behavior in response to mechanical stimuli other than ultrasound, we treated proteoliposomes with lysophosphotidylcholine (LPC) which is a functional assay and another stimulus in addition to ultrasound. LPC has been reported to gate MscL by changing the membrane lateral tension profile surrounding the protein channel^40^. The fluorescent signal was monitored in control-vesicles, and in liposomes reconstituted with MscL, NaK 2K F92A or KvAP channels for 5 min and 30 min before and after LPC treatment respectively. Liposomes were then lysed using 0.5% Triton-X 100, followed by a 5 min signal measurement to release all liposome-encased calcein. All signals were normalized to the total florescent value measured after triton treatment.

### Statistical Analyses

LPC data were processed using Graphpad Prism software. The statistical tests were conducted using Mann-Whitney test.

Ultrasound modulation results were analyzed statistically with Stata software (StataCorp LP, College station, Texas), using mixed effects linear regression model with date random intercept. In duty cycle and duration studies, statistical analysis was performed comparing varying parameters within each group separately (control vesicle and MscL). Similar statistical analysis was performed on the data collected from the protein concentration variation effect on LIFU modulation. Different concentrations were compared to one another and to control vesicles per sample group (MscL, NaK2K F92A, KvAP). The statistical significance *p*-value is presented as followed: *p < 0.05, **p < 0.01; ***p < 0.001).
